# Pharmacotherapy during Pregnancy, Childbirth, and Lactation

**DOI:** 10.3390/ijerph191811336

**Published:** 2022-09-09

**Authors:** Karel Allegaert

**Affiliations:** 1 Child and Youth Institute, KU Leuven, Herestraat 49, 3000 Leuven, Belgium; karel.allegaert@uzleuven.be; Tel.: +32-(0)-1634-2020; 2 Department of Development and Regeneration, KU Leuven, 3000 Leuven, Belgium; 3 Department of Pharmaceutical and Pharmacological Sciences, KU Leuven, 3000 Leuven, Belgium; 4 Department of Hospital Pharmacy, Erasmus Medical Center, 3000 GA Rotterdam, The Netherlands

Pharmacotherapy is a very powerful approach to truly improve outcomes for pregnant women and their newborns. Associated with changes in the clinical characteristics of pregnant women (age, weight, co-morbidity), the extent of medical utilization and herbal medicine use in pregnancy has substantially increased. However, as little as 5% of available medicines have been properly monitored, tested and labeled for use in pregnancy and lactation. Pregnant or lactating women are usually excluded from clinical trials, while product development for diseases specific to pregnancy or perinatal—including fetal—indications is very limited. However, in real life, pregnant women get ill, and women with underlying medical conditions get pregnant. As a consequence, individual pregnant or lactating women and their caregivers are left in the dark about risks they cannot oversee, and they need to take responsibility for this uncertainty. The risks of pharmacotherapy are dual in pregnancy, both for the pregnant woman and her fetus. Pregnancy-related alterations in either pharmacokinetics and/or pharmacodynamics may lead to inadequate therapeutic response or maternal toxicity (and subsequent also fetal risks), while maternal–fetal transmission may impair fetal organ formation, formation or even cause fetal demise.

Pharmacokinetic changes relate to altered gastric motility, increase in body weight, fat deposition, or plasma volume and an associated decrease in albumin. Medicines’ metabolizing enzyme activity alters in both directions (either increase, or decrease), there is an increase in hepatic and renal blood flow, and a mirrored increase in glomerular filtration rate [1]. Acetaminophen pharmacokinetics, the impact of pregnancy, and the research tools to explore these changes have recently been described, and serve as an illustration of the extent and impact [2]. Compared to classic non-compartmental analyses, analyses applying either population pharmacokinetic modeling or physiologically based pharmacokinetic (PBPK) modeling are much more advanced, while PBPK can also inform us about placental and fetal exposure [3]. We refer interested readers to some recent reviews on this topic [4,5,6].

Pharmacodynamics refers to, e.g., glucose tolerance, hemodynamics and pregnancy-related blood pressure changes, or changes in immunological function (e.g., auto-immune diseases, or infectious diseases) [1].

These modeling software programs are only one of the urgently needed tools for more evidence-based pharmacotherapy. At best, this should be driven by a balanced, data-informed decision between disease-related risks (natural course during pregnancy) and potential risks related to exposure to medicines for the mother, fetus or infant. Awareness of this discrepancy, and knowledge gaps in pregnant and lactating women, stimulated authorities, such as the Food and Drug Administration (FDA) and the European Medicines Agency (EMA), to further develop this clinical research field. This includes, e.g., the Pregnancy and Lactation Labeling Final Rule, FDA guidance on inclusion in clinical trials [7], or EMA guidelines on good pharmacovigilance practice in this specific population [8].

In the broader clinical research community, this concern is addressed by projects such as the ‘Drugs in Lactation’ Analysis Consortium (DLAC, recently finalized) [9], the Obstetric Pharmacology Research Unit Network (related to the Eunice Kennedy Schriver Institute, National Institute of Child Health and Human Development (NICHD)) [10], or the Innovative Medicines Initiative (IMI) Conception project (https://www.imi-conception.eu/, accessed on 29 August 2022). Alternatively, there are projects with a specific focus on, e.g., Human Immunodeficiency Virus (HIV) (pharmacokinetics of newly developed antiretroviral agents in HIV-infected pregnant women, PANNA) [11], or in transplanted women who are pregnant [12].

Within this dynamic environment, and in an attempt to increase awareness and scientific output, the first Special Issue entitled ‘Pharmacotherapy During Pregnancy, Childbirth and Lactation’ of the *International Journal of Environmental Research and Public Health* has been launched. Because of the relevance of the topic, we are hereby proud to announce that a second Special Issue on the same topic will be launched in the coming weeks.

When reflecting on the topics discussed in the current issue, we can discriminate four different research lines or themes: (1) prescription practices, and their covariates, (2) illustrations on pharmacovigilance, (3) explorations of knowledge skills or information needs of different stakeholders, and finally, (4) true novelties that are promising approaches, and the issues and thresholds encountered during the drug development plans. We have further summarized these themes, with reference to the specific papers included in the Special Issue in Figure 1.

The ***first theme*** focused on prescription and medicine utilization patterns. D’Aloja et al. reported on the prescription pattern of medicines among pregnant immigrant women from countries with high migratory pressure (HMPCs) compared to pregnant Italian women. As the migratory phenomenon represents a structural element of society, it is hereby reassuring that the prescription patterns only showed minor differences between both groups, while inappropriate prescription (antihypertensives, statins, anti-inflammatory drugs) was limited in both groups [13]. Two other papers focused on another ‘intercurrent’ event (the COVID-19 pandemic) and its effects on medicines utilization in pregnant and breastfeeding women in different European countries [14,15]. Both studies hereby used a self-reported approach of the end users. In a cross-sectional study (Ireland, Norway, Switzerland, the Netherlands and the United Kingdom), perinatal medicines use during the first pandemic wave (June–July 2020). The general pattern confirmed the high prevalence of medicine use (60% used at least one medicine in the last 3 months), with some differences related to country, maternal age, professional status or time since delivery, as well as the presence of chronic or acute (COVID-19 screening) diseases [14]. In a subsequent analysis, during the third wave (June–August 2021) in the same countries, 41.4% used at least one medicine, with analgesics (paracetamol), systemic antihistamines (cetirizine), and medicines for gastric disorders (omeprazole) were the three most commonly used. Anti-infectives were less prevalent compared to the pre-pandemic setting, while the use of antidepressants and anxiety-related medicines remained similar [15]. The two other papers related to the prescription theme have a specific focus on young women with specific medical conditions. In a cross-sectional study of 380 women using combined oral contraceptives, category 3–4 contraindications were present in 31.3% and included—among others—controlled hypertension, major surgery with immobilization, migraine with aura, breastfeeding, or diabetes mellitus with complications [16]. The use of prescribed medicines to treat chronic diseases during pregnancy has been assessed, using the Swiss Health Care claims data for reimbursement [17]. Overall, patterns of claims for medicines to treat chronic diseases during pregnancy in Switzerland were in line with treatment recommendations, but rare events of in utero exposure to teratogenic medicines (like renin-angiotensin-aldosterone system inhibitors, or some immune-suppressants). Levothyroxine (6.6%) was the most frequently claimed medicine, antihypertensives (5.3%) were second (nifedipine 3.9%), insulin (3.3%), psychotropics (3.8%) or medicines for obstructive airway diseases (3.6%) were also common [17].

The ***second theme*** reflects on illustrations of perinatal pharmacovigilance management and efforts to improve our practices. Knowledge of the impact of in utero exposure to lithium and how to address this during the postnatal period is still limited. Torfs et al. described the postnatal management in 10 neonates, and provided a postnatal care protocol to enhance the quality of neonatal care, and guide parental counseling [18]. Tajima et al. illustrated that administrative data to identify birth-related outcomes with the end date of pregnancy is another effort to improve our practices, and to create useful and accessible tools to generate information from already available information [19]. Finally, and using a systematic review approach, the risk for neonatal hypoglycemia and bradycardia after maternal beta-blocker use has been quantified [20]. Based on this meta-analysis, the authors concluded that there is a probable risk of hypoglycemia (certainty of evidence: Moderate) and possible risk of bradycardia (certainty of evidence: Low) in neonates upon fetal beta-blocker exposure [20]. Consequently, it was suggested to monitor glycemia and consider monitoring the heart rate in the first 24 h of life [20].

The ***third theme*** considered explorations on knowledge skills and information needs of different stakeholders involved in pharmacotherapy. First, a questionnaire to assess the knowledge of midwives and pediatric nurses on maternal use of analgesics was developed. This questionnaire was subsequently pilot tested to validate (construct validity) this tool and is made available for research and educational purposes [21]. Although marketing authorization holders (MAHs) are involved in monitoring medication safety, it was unclear how they experience their role and current monitoring activities in pregnancy. A qualitative study (online focus groups with 38 representatives of nine organizations) identified multiple difficulties with data collection and conflicts inherent to MAHs’ data processing (mistrust, legal obligations and regulatory framework, and being ‘outside’ the healthcare context) [22]. MAHs suggested that data registration should occur in close collaboration with patients and healthcare professionals organized within the healthcare context and performed by using a user-friendly system [22]. To collect such data, we also need to understand the information needs and counseling preferences of potential users (public survey respondents or health care professionals) of a Teratology Information Service (TIS) [23].

Finally, the ***fourth theme*** provided some future perspectives on new targets to consider, or promising approaches to issues and thresholds encountered during the drug development plan. Broekhuizen et al. described their experience and data on the kynurenine pathway in the placenta as a potential novel pharmacotherapeutic target. This includes a detailed description of how the placental kynurenine pathway is altered in pregnancy complications, including recurrent miscarriage, preterm birth, preeclampsia, and fetal growth restriction. Importantly, the authors described that these alterations do not affect maternally circulating metabolite concentrations, suggesting that the kynurenine pathway functions as a local signaling—placenta-related—pathway [24]. The higher incidence of neonatal pulmonary hypertension in the sildenafil arm of the Dutch STRIDER trial for severe fetal growth restriction resulted in a very and likely too cautious reaction by many competent authorities, as halting any prenatal sildenafil use [26]. De Bie et al. summarize the indications for sildenafil use during the second and third trimesters of pregnancy [25]. These include maternal pulmonary hypertension, preeclampsia, preterm labor, fetal growth restriction, oligohydramnios, fetal distress, and congenital diaphragmatic hernia [25]. For most indications, the rationale for administering prenatal sildenafil is based on limited, equivocal data from in vitro studies and rodent disease models, and is specific for congenital diaphragmatic hernias [25].

In conclusion, this Special Issue has highlighted recent progress and knowledge gaps that should be addressed to further improve pregnancy and lactation-related pharmacotherapy. Furthermore, new tools, new approaches and new targets were discussed, reflecting also the dynamics of the specific research field. We hope that this Special Issue provides the reader with state-of-the-art reviews on various aspects of perinatal pharmacology. We hope that these efforts boost collaboration to further shift from best-guess practices to evidence-based knowledge on pharmacotherapy for pregnant women, their fetuses and newborns.

## Figures and Tables

**Figure 1:**
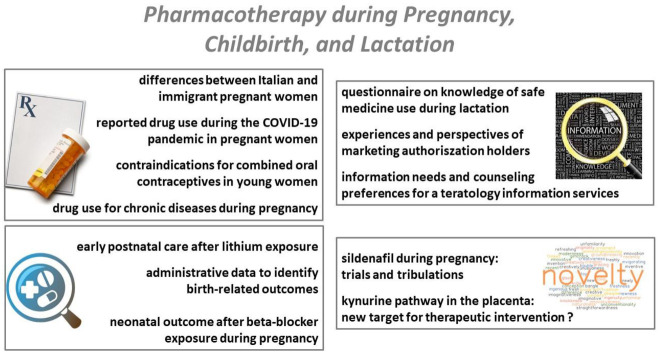
Overview on the different themes and specific papers discussed in this Special Issue [13,14,15,16,17,18,19,20,21,22,23,24,25].
